# Left Main Coronary Artery Aneurysm

**Published:** 2016-01-13

**Authors:** Hossein Doustkami, Nasrollah Maleki, Zahra Tavosi

**Affiliations:** 1 *Imam Khomeini Hospital, Ardabil University of Medical Sciences, Ardabil, Iran. *; 2 *Khalije Fars Hospital, Bushehr University of Medical Sciences, Bushehr, Iran. *

**Keywords:** *Coronary vessels*, *Coronary angiography*, *Aneurysm*

## Abstract

Aneurysms of the left main coronary artery are exceedingly rare clinical entities, encountered incidentally in approximately 0.1% of patients who undergo routine angiography. The most common cause of coronary artery aneurysms is atherosclerosis. Angiography is the gold standard for diagnosis and treatment. Depending on the severity of the coexisting coronary stenosis, patients with left main coronary artery aneurysms can be effectively managed either surgically or pharmacologically. We herein report a case of left main coronary artery aneurysm in a 72-year-old man with a prior history of hypertension presenting to our hospital because of unstable angina. The electrocardiogram showed ST-segment depression and T-wave inversion in the precordial leads. All the data of blood chemistry were normal. Echocardiography showed akinetic anterior wall, septum, and apex, mild mitral regurgitation and ejection fraction of 45%. Coronary angiography revealed a saccular aneurysm of the left main coronary artery with significant stenosis in the left anterior descending, left circumflex, and right coronary artery. The patient immediately underwent coronary artery bypass grafting and ligation of the aneurysm. At six months’ follow-up, he remained asymptomatic.

## Introduction

Coronary artery aneurysms (CAAs) are an uncommon disease defined as dilated segments larger than 1.5 times the diameter of the adjacent coronary arteries.^1^ The aneurysms occur most often in the right coronary artery, followed in frequency by the circumflex and anterior descending arteries. Aneurysms of the left main coronary artery are very rare clinical entities,^2^ the most common cause being atherosclerosis, which accounts for at least half of the CAAs diagnosed by pathology or angiography in adults.^3^ Because CAAs are usually found incidentally during cardiac examinations, most of the reported cases have been diagnosed via coronary angiography, intravascular ultrasound and, on rare occasions, during autopsy. 

Here we describe a 72-year-old man presenting with unstable angina whose coronary angiogram revealed a saccular aneurysm of the left main coronary artery.

## Case Report

**Figure 1:**
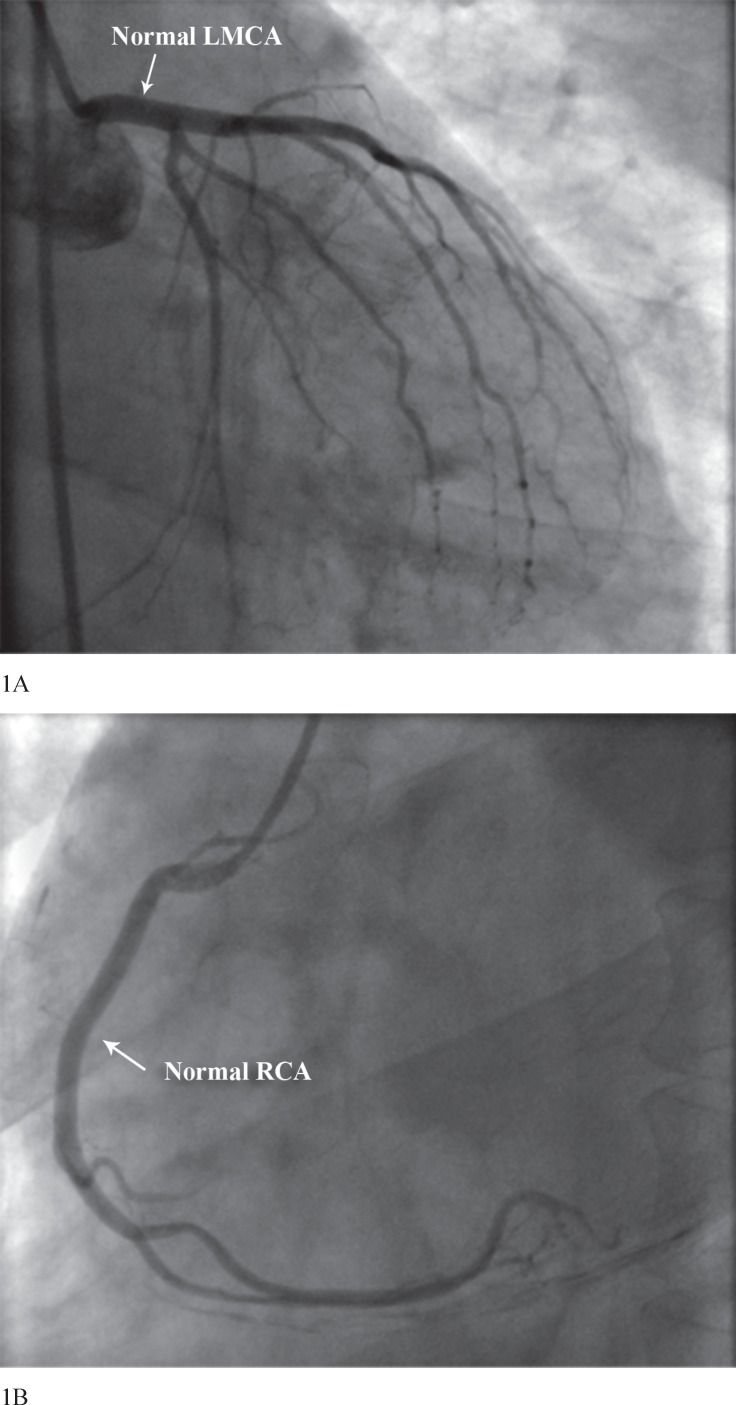
Right anterior oblique coronary angiographic view (A), showing a normal left main coronary artery (LMCA). Left anterior oblique coronary angiographic image (B), showing a normal right coronary artery (RCA)

A 72-year-old man with a prior history of hypertension presented to our hospital in April 2013 owing to the acute onset of chest pain at rest, which radiated to his jaw and left arm and was associated with profuse sweating. He denied any history of chest trauma, syphilis, pericarditis, or previous myocardial infarction. The patient had undergone coronary angiography in July 2001 because of angina pectoris, which revealed normal coronaries with a normal left main anatomy (Figure 1). On examination, he had a pulse of 92/minute, blood pressure of 170/90 mmHg, respiratory rate of 20/minute, and temperature of 37.1 °C. Palpation of the peripheral pulses and of the precordium, inspection of the jugular veins, and auscultation of the lungs disclosed normal findings. There were no peripheral arterial bruits. Cardiac examination revealed normal first and second heart sounds. There was an atrial gallop sound and a grade 2/6 systolic ejection murmur along the left sternal border. 

All the data of blood chemistry were normal. Electrocardiography showed ST-segment depression of 2 mm and T-wave inversion in the precordial leads. Chest radiography was normal. Transthoracic echocardiography showed akinetic anterior wall, septum, and apex, mild mitral regurgitation, and ejection fraction of 45%. The patient improved with bed rest, aspirin, clopidogrel, statin, beta-blocker, intravenous nitroglycerin, and anticoagulation.

**Figure 2:**
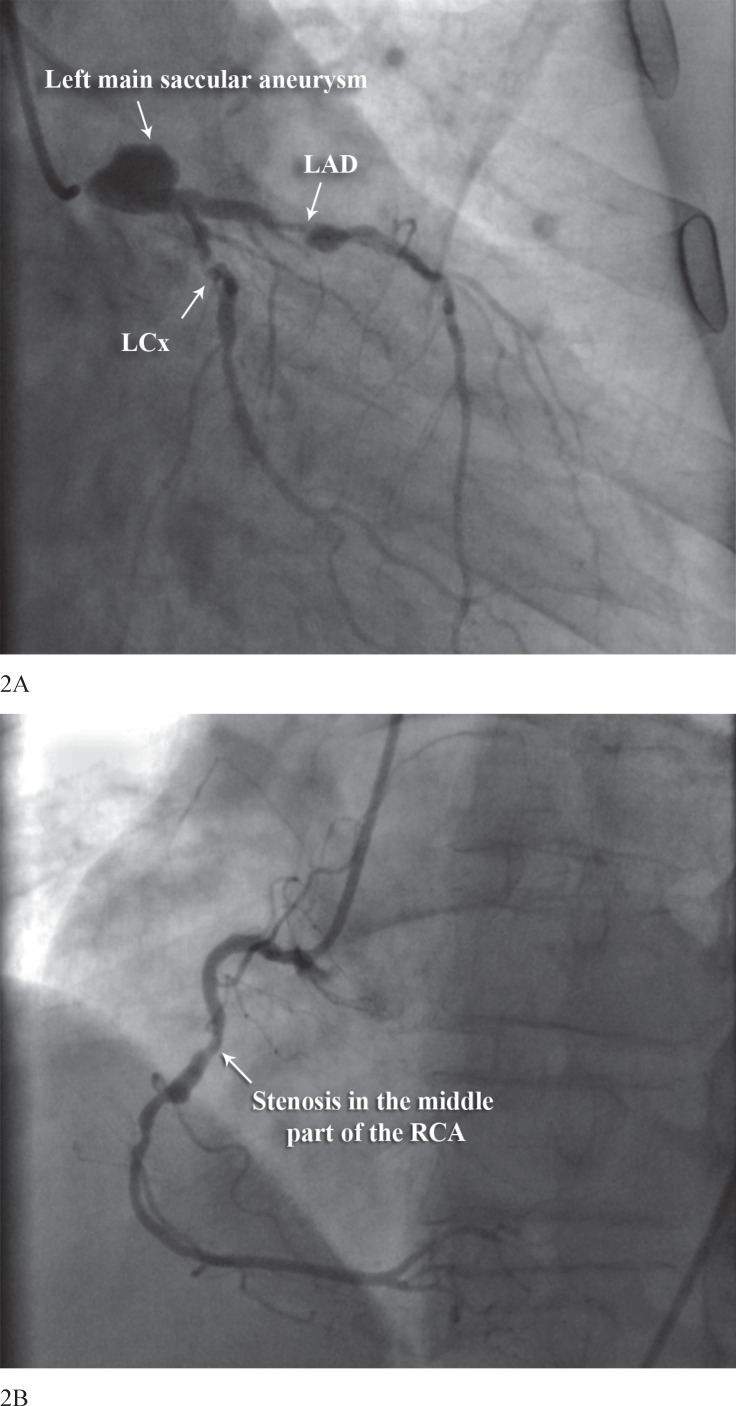
Right anterior oblique coronary angiographic view (A), showing a saccular aneurysm of the left main coronary artery with 90% stenosis in the proximal portion of the left anterior descending (LAD) and, 99% stenosis in the left circumflex (LCx) arteries. Left anterior oblique coronary angiographic image (B), showing an 80% stenosis in the middle part of the right coronary artery (RCA)

Coronary angiography revealed a saccular aneurysm of the left main coronary artery, measuring 21 × 18 mm with 90% stenosis in the proximal portion of the left anterior descending, 99% stenosis in the proximal portion of the left circumflex, and 80% stenosis in the middle part of the right coronary artery (Figure 2). The patient immediately underwent coronary artery bypass grafting and ligation of the aneurysm on cardiopulmonary bypass. The ascending aorta was opened, and the orifice of the left coronary artery was oversewn and closed with 3-0 polypropylene, and the distal portion of the left main coronary artery was ligated. The patient had an uneventful hospitalization and was discharged on aspirin and warfarin therapy. At 6 months’ follow-up, he remained asymptomatic.

## Discussion

The first pathologic description of a CAA was published by Morgagni^4^ (1761), and the first clinical case of an aneurysm was reported by Bourgon^5^ (1812), who described a post-mortem finding of a dilatation in the right coronary in a patient who had suffered sudden death. Morphologically, these aneurysms may be saccular or fusiform, single or multiple. The histopathological features of CAAs are based on the destruction of the media of the arterial wall, and this thinning of the media together with increased wall stress causes progressive dilatation of that segment of the coronary artery. Aneurysms are often classified by their macroscopic shape and size, according to the transversal and longitudinal size. Saccular aneurysms are spherical and show a transverse diameter greater than the longitudinal diameter, whereas fusiform aneurysms are characterized by a gradual and progressive dilatation that involves the complete circumference of the artery and have a transverse diameter that is smaller than the longitudinal diameter.^6^

The incidence of CAAs ranges widely from 0.3% to 5.3% of the population, with pooled data showing a mean incidence rate of 1.65%.^3^ There is a positive correlation between the incidence of aneurysms in major coronary arteries and their size and bag-like shape.^7^ The largest autopsy series to date, by Daoud et al.,^8^ showed a 1.4% incidence rate of CAAs in 694 patients. In the CASS (Coronary Artery Surgery Study) registry, the angiographic incidence of this abnormality was 4.9% in a group of 20 087 patients.^9^ CAAs are said to be giant when the size is more than 8 mm. Left main CAAs are the most uncommon coronary abnormality among all CAAs. The largest published series of left main CAAs is that of Topaz and coworkers,^10^ who reported 22 cases. The incidence of left main CAAs in that study was 0.1% among 20 332 adult patients who underwent routine coronary angiography. The width of the largest left main CAA observed by this group was 1.9 cm. In a study of 3200 coronary angiographies,^11^ 22 patients were identified with CAAs (0.68%). The aneurysms were found at the stem (12%), anterior descending (52%), right coronary artery (20%), and circumflex artery (16%). Table 1 shows exclusively the CAA prevalence in the most recent epidemiologic studies.

The most common cause of CAAs is atherosclerosis, which accounts for at least half of the CAAs diagnosed by pathology or angiography in adults.^1^ Other causes of CAAs include congenital malformation, Kawasaki disease, traumatic injury, previous balloon angioplasty, sub-acute bacterial endocarditis, rheumatic fever, mycosis, syphilis, polyarteritis nodosa, systemic lupus erythematosus, Ehlers-Danlos syndrome, scleroderma, Marfan syndrome, Takayasu arteritis, and genetic syndromes (Loeys-Dietz syndrome).^12^^, ^^13^

In most cases, CAAs are asymptomatic; then when symptomatic, the clinical manifestations depend on the underlying cause. Although CAAs can be seen at any age, those related to atherosclerosis usually appear later in life than those of a congenital or inflammatory nature. No clinical characteristics exist for atherosclerotic CAAs. The clinical manifestations are similar to those seen in coronary artery disease,^14^ and the patients may have angina pectoris, dyspnea, edema, myocardial infarction, and sudden death.^15^ Occasionally, a systolic murmur is heard over the precordium. An association with an abdominal aortic aneurysm and hypertension can occur. Patients with coronary ectasias seem to suffer more frequently from myocardial infarctions than do patients with only stenotic coronary heart disease.^16^ Large aneurysms, particularly those that are partially or completely filled with mural thrombi, may create diagnostic challenges by masquerading as cardiac masses.^17^ The main differential diagnoses include pericardial cysts and primary and metastatic tumors.^14^ The natural history of this condition is not known.^18^

**Table 1:** Prevalence of coronary artery aneurysms in angiographic and autopsy studies

Source	Year	Diagnostic Method	Population	Prevalence (%)
Falsetti & Carrol^7^	1976	Angiography	742	1.5
Daoud et al.^7^	1983	Autopsy	694	1.4
Tunick et al.^13^	1990	Angiography	8422	0.2
Wang et al.^1^	1999	Angiography	10120	0.1
Harikrishnan et al.^10^	2000	Angiography	3200	0.7
Groenke et al.^16^	2005	Angiography	7101	1.4
Rozenberg et al.^18^	2005	Autopsy	1000	1.5

CAAs may be detected by noninvasive tools, including echocardiography, computed tomography, and magnetic resonance imaging. Nevertheless, coronary angiography remains the best method for the assessment of coronary anatomy and pathology.^19^ Coronary angiography provides additional information regarding the size, shape, location, and number of the existing anomalies and shows an image of the coronary artery status,^20^ determining the extent and severity of the coronary lumen obstruction in coronary artery disease.^21^ Undoubtedly, invasive coronary angiography remains the gold standard for the evaluation of CAAs for a number of reasons. Only the blood flow within the lumen can be evaluated, and conventional invasive coronary angiography provides no information about the vessel wall. Thus, with conventional coronary angiography, the true size of the aneurysm may be underestimated or the aneurysm may not even be seen when it is occluded or contains substantial thrombi or plaque.^22^

CAA treatment consists of medical management, surgical resection, and stent placement; however, the appropriate treatment for CAAs is controversial and depends on the particular clinical situation. The recently available results have been based primarily on case reports and not on controlled studies, which continues to cause a therapeutic dilemma. The medically conservative therapy generally consists of attempts to prevent thromboembolic complications in patients with aneurysmal arteries who are at increased thrombotic risk through the administration of antiplatelet and anticoagulant medication.^23^

Surgical management is appropriate in symptomatic patients who have obstructive coronary artery disease or evidence of embolization leading to myocardial ischemia and in patients with CAAs with a risk of rupture. Surgery is also indicated in cases of progressive left main coronary artery enlargement as documented by serial angiographic measurement.^22^ Recently, percutaneous application of polytetrafluoroethylene (PTFE)-covered stents has gained popularity due their ability to effectively limit the expansion of CAAs by reducing the blood flow within the aneurysm, thereby preventing their rupture. Some authors have suggested that PTFE-covered stents should be limited to patients whose aneurysms are smaller than 10 mm in diameter.^24^ Percutaneous strategies also include coil embolization, autologous saphenous vein-covered stent grafting, and one case report of drug-eluting stent implantation superimposed on a PTFE-covered stent graft.^25^ Surgical strategies that have been described include aneurysm ligation, resection, marsupialization with interposition graft, and coronary artery bypass surgery.

## Conclusion

CAAs are an uncommon and often accidental finding. CAAs are usually associated with atherosclerosis in adults in Western countries. Angiography is the gold standard for diagnosis. The optimal therapy for patients with CAAs is unknown, and controversy persists regarding the use of medical or surgical modalities.

## Notes:

***This paper should be cited as:*** Doustkami H, Maleki N, Tavosi Z. Left Main Coronary Artery Aneurysm. J Teh Univ Heart Ctr 2016;11(1):41-45.
